# The Wine Ecosystem as a Reservoir for Potential Probiotics: A Comparative In Vitro Evaluation of *Lactiplantibacillus plantarum* and *Oenococcus oeni* Isolates

**DOI:** 10.3390/foods15061025

**Published:** 2026-03-15

**Authors:** Chong Yuan, Yuanyuan Liu, Gongchen He, Tongxin Xu, Ping Wang, Jingyue Liu, Shuwen Liu, Kan Shi

**Affiliations:** Hainan Institute of Northwest A&F University, College of Enology, Heyang Experimental and Demonstrational Stations for Grape, Ningxia Helan Mountain’s East Foothill Wine Experiment and Demonstration Station, Northwest A&F University, Yangling 712100, China

**Keywords:** *Lactiplantibacillus plantarum*, *Oenococcus oeni*, probiotic screening, wine ecosystem, microbiota reservoir

## Abstract

The wine ecosystem constitutes a highly selective ecological niche characterized by low pH, high ethanol levels, sulfur dioxide, polyphenols, and nutrient limitation. During malolactic fermentation, this environment becomes dominated by specialized lactic acid bacteria (LAB), particularly *Lactiplantibacillus plantarum* and *Oenococcus oeni*, whose persistence under such stressors suggests the presence of adaptive traits relevant to probiotic development. In this study, twenty-three LAB isolates obtained from the spontaneous wine ecosystem were systematically evaluated through a multi-stage screening strategy. Primary single-factor assays revealed pronounced inter- and intraspecies variability in tolerance to acid, lysozyme, and bile salts. As a result, all *O. oeni* isolates and eight *L. plantarum* strains were excluded from further consideration. The four selected *L. plantarum* isolates (M-1, SY-2, XJA2, and XJ14) were subsequently subjected to simulated gastrointestinal challenges. Strains M-1 and XJ14 maintained high viability across both gastric and intestinal phases. In contrast, SY-2 and XJA2 exhibited pronounced gastric sensitivity but demonstrated strong survival in the intestinal phase. Functional characterization further distinguished the isolates: M-1 and XJ14 displayed balanced probiotic profiles, whereas XJA2 exhibited exceptional auto-aggregation and efficient metabolic capacity, suggesting specific colonization potential despite its gastric vulnerability. Comprehensive safety assessments confirmed the absence of hemolytic activity, biogenic amine production, and acquired antibiotic resistance in the tested isolates. Collectively, these findings identify M-1 and XJ14 as promising candidates for direct probiotic application, and XJA2 as a promising functional strain for encapsulation-based delivery. This study highlights the wine ecosystem as a valuable reservoir for novel probiotic development.

## 1. Introduction

Probiotics are defined as “live microorganisms which, when administered in adequate amounts, confer a health benefit on the host”. Lactic acid bacteria (LAB) represent one of the most widely recognized probiotic groups, valued for their extensive presence in fermented foods and their well-documented roles in regulating gut microbiota [1], enhancing epithelial barrier integrity [2], and modulating host immune responses [3]. Driven by increasing consumer awareness of gut health, the global probiotics market has experienced substantial economic growth, with widespread applications in the functional food industry [4]. These beneficial microbes are of profound interest to consumers seeking preventative health measures, as well as to patients requiring therapeutic dietary interventions for gastrointestinal disorders, such as irritable bowel syndrome and gut dysbiosis [5,6]. Furthermore, there is a growing consumer demand for non-dairy, plant-based probiotic alternatives suitable for lactose-intolerant or vegan populations. While commercial probiotics are predominantly derived from dairy or human intestinal sources, non-dairy fermentation systems, such as plant-based matrices [7], are increasingly recognized as important reservoirs for novel probiotic candidates, driven by their unique microbial diversity and the robust selective pressures exerted by these environments.

The evaluation of potential probiotic strains typically follows a hierarchical framework comprising three essential components: prerequisite survival, functional attributes, and safety assurance. First, a strain must demonstrate the fundamental capacity to withstand the harsh physiological barriers encountered during gastrointestinal (GIT) transit, including salivary lysozyme, gastric acidity, bile salts, and digestive enzymes [8]. These survival traits determine whether cells can reach the intestine alive and therefore qualify for further functional characterization. Second, strains that successfully endure GIT-like stressors must possess key functional properties. Among these, adhesion and colonization potential are particularly crucial, as sustained residence within the intestine is often required for the exertion of health-promoting effects [9]. Adhesive capacity can be inferred in vitro from traits such as auto-aggregation and cell-surface hydrophobicity [10]. Additional functional indicators include the ability to produce beneficial metabolites, including organic acids or antimicrobial compounds, and the capacity to utilize dietary prebiotics such as oligosaccharides, reflecting metabolic adaptability and potential synbiotic compatibility [11,12]. Finally, rigorous safety evaluation is indispensable. Candidate strains must exhibit no hemolytic activity, must not produce harmful metabolites such as biogenic amines, and must lack acquired antibiotic resistance determinants [13,14]. Together, these criteria ensure that only strains with robust survival, meaningful functionality, and confirmed biosafety are considered credible probiotic candidates.

The wine ecosystem constitutes a distinctive ecological niche characterized by multiple selective pressures, including low pH (typically 3.2–3.8), elevated ethanol concentrations, antimicrobial constituents such as sulfur dioxide and polyphenols, and limited nutrient availability. LAB species that persist under such conditions, primarily *Lactiplantibacillus plantarum* and *Oenococcus oeni*, have evolved strong stress-response mechanisms [15]. Notably, many of these stressors parallel the hostile conditions encountered during human GIT transit. This ecological similarity suggests that wine-derived LAB may possess inherent tolerance to GIT-like stresses, making them promising candidates for the development of novel probiotics.

Although studies investigating the probiotic potential of wine-derived LAB remain limited, available evidence is encouraging. For instance, García-Ruiz et al. [15] reported that *Pediococcus* and *Lactobacillus* strains isolated from wine exhibited strong tolerance to simulated GIT environments, in some cases outperforming commercial reference strains. Likewise, Su et al. [16] demonstrated that certain *O. oeni* strains exerted measurable anti-inflammatory effects in vitro. However, published data regarding species- and strain-level differences in resistance to specific gastrointestinal barriers remain scarce and, in some cases, inconsistent. These knowledge gaps highlight the need for systematic, multi-stage comparative evaluations of wine-derived LAB to clarify interspecies and intraspecies variation in stress resilience and probiotic-relevant functional traits.

Therefore, this study aimed to conduct a systematic and comparative in vitro screening of LAB isolated from the Chinese wine ecosystem. The primary objective was to evaluate and contrast the probiotic potential of *L. plantarum* and *O. oeni* using a stepwise filtering strategy designed to identify the most resilient strains. This “screening funnel” approach comprised: (1) primary evaluation of tolerance to single stress factors (low pH, lysozyme, and bile salts); (2) secondary assessment of survival under complex simulated gastrointestinal conditions (SGJ and SIJ); and (3) tertiary characterization of functional properties (auto-aggregation, prebiotic utilization) together with essential safety indicators (hemolytic activity, biogenic amine production, and antibiotic susceptibility).

## 2. Materials and Methods

### 2.1. Strains and Culture Conditions

A total of 23 lactic acid bacteria (LAB) strains were used in this study, comprising 12 *L. plantarum* and 11 *O. oeni* strains. These strains were previously isolated by the laboratory from the malolactic fermentation (MLF) of wines made from indigenous Chinese grape varieties and were identified by partial 16S rRNA gene sequencing (amplified fragment lengths of 1025 bp for *O. oeni* and 318 bp for *L. plantarum*) with a similarity threshold of >97%. A reference probiotic strain, *L. plantarum* ATCC 14917, was included for comparative analysis. All reference strains were obtained from our laboratory collection.

*L. plantarum* strains and the reference strain were cultured statically under facultative anaerobic conditions at 37 °C in MRS broth (10 g/L proteose peptone, 10 g/L beef extract, 4 g/L yeast extract, 20 g/L glucose, 1 mL/L Tween-80, 2 g/L dipotassium hydrogen phosphate, 2 g/L diammonium citrate, 5 g/L sodium acetate, 0.2 g/L MgSO_4_·7H_2_O, 0.05 g/L MnSO_4_·H_2_O, pH 6.0). *O. oeni* strains were cultured statically under facultative anaerobic conditions at 26 °C in ATB broth (10 g/L glucose, 5 g/L yeast extract, 10 g/L peptone, 0.2 g/L MgSO_4_·7H_2_O, 0.05 g/L MnSO_4_·H_2_O, 0.5 g/L cysteine-HCl, and 250 mL/L tomato juice, pH 4.8). The pH of all media was adjusted to the respective target values prior to sterilization. All stock cultures were stored at −80 °C in their respective broths supplemented with 50% (*v*/*v*) glycerol (Solarbio, Beijing, China). All strains were sub-cultured twice before experimental use to ensure metabolic activity.

### 2.2. Primary Screening: Single-Factor Stress Tolerance

The primary screening was structured as a sequential funnel to streamline the selection process. Strains were tested one after another in the order of acid, lysozyme, and bile salt stresses. Strains that exhibited extreme sensitivity (survival < 25%) to a preceding stressor were immediately excluded from subsequent single-factor assays. For all stress assays, bacterial cultures were grown to the stationary phase (OD_600_ ≈ 1.0). Cells were harvested by centrifugation (MIKRO-200R, Hettich, Tuttlingen, Germany) at 6000× *g* for 8 min, washed three times with sterile 0.85% (*w*/*v*) saline, and resuspended in 1 mL of saline before being introduced to the stress media. All stress tolerance assays were performed using three independent biological replicates. For viability determination, the raw colony-forming unit (CFU/mL) data were log10-transformed before calculating the survival percentages.

#### 2.2.1. Tolerance to Acid Stress

Acid tolerance was assessed using the method of Huang et al. [17] with modifications. Stationary-phase cell suspensions were inoculated into MRS broth previously adjusted to pH 3.2 with HCl using a pH meter (PB-10, Sartorius, Göttingen, Germany). After incubation at 37 °C for 5 h, bacterial viability at 0 h and 5 h was determined using the plate count technique.

#### 2.2.2. Tolerance to Lysozyme Stress

Lysozyme tolerance was assessed following the method of Zago et al. [18] with modifications. Harvested cells were resuspended in Ringer’s solution (8.5 g/L NaCl, 0.4 g/L KCl, 0.34 g/L CaCl_2_) and inoculated into sterile electrolyte solution (SES; 0.22 g/L CaCl_2_, 6.2 g/L NaCl, 2.2 g/L KCl, 1.2 g/L NaHCO_3_) containing 100 mg/L filter-sterilized (0.22 µm) lysozyme (Solarbio, Beijing, China) to simulate saliva dilution. Samples were incubated at 37 °C, and viable counts were performed by spread-plating on their respective optimal media (MRS agar for *L. plantarum* and ATB agar for *O. oeni*) at 0, 0.5, and 2 h. The survival rate was expressed as the proportion of viable counts remaining after incubation relative to the initial (0 h) count.

#### 2.2.3. Tolerance to Bile Salt Stress

Bile tolerance was examined based on the method of Vera-Pingitore et al. [19] with modifications. Cell suspensions were inoculated (1:10 *v*/*v*) into MRS broth supplemented with 0.3% (*w*/*v*) bile salts (Solarbio, Beijing, China). Following incubation at 37 °C for 2 h, samples were plated at 0 h and 2 h to determine viable counts. The survival rate was determined by comparing the final colony counts to the initial counts.

### 2.3. Secondary Screening: Simulated Gastrointestinal Tract (GIT) Tolerance

Based on the results of the primary screening, four *L. plantarum* strains (M-1, SY-2, XJA2, and XJ14) that demonstrated concurrent tolerance to acid, lysozyme, and bile salts were further subjected to simulated gastrointestinal conditions.

#### 2.3.1. Simulated Gastric Juice (SGJ) Tolerance

Referencing the method of Vasiee et al. [20] with slight modifications, SGJ was prepared with 7.31 g/L NaCl, 0.52 g/L KCl, 3.78 g/L NaHCO_3_, and 2 g/L pepsin (activity ≥ 250 U/mg; Solarbio, Beijing, China). The presence of NaHCO_3_ simulates the natural ionic and buffering properties of gastric fluid. The solution was adjusted to pH 3.0 and filter-sterilized. Harvested cells were resuspended in the SGJ (1:10 *v*/*v*), incubated at 37 °C for 3 h, and then plated. The survival rate was calculated by comparing the viable counts at 3 h to those at 0 h.

#### 2.3.2. Simulated Intestinal Juice (SIJ) Tolerance

Referencing the method of Vasiee et al. [20] with slight modifications, SIJ was prepared by dissolving 250 mg/L pancreatin (from porcine pancreas; protease ≥ 100 U/mg, amylase ≥ 400 U/mg, lipase ≥ 20 U/mg; Solarbio, Beijing, China) and 0.45% (*w*/*v*) porcine bile extract in 0.5% (*w*/*v*) sterile saline. The solution was adjusted to pH 8.0 and filter-sterilized. Harvested cells were resuspended in the SIJ (1:10 *v*/*v*), incubated at 37 °C for 4 h, and then plated. The survival rate was calculated by comparing the viable counts at 4 h to those at 0 h.

### 2.4. Tertiary Screening: Functional and Safety Characterization

#### 2.4.1. Assessment of Auto-Aggregation Ability

The auto-aggregation ability of LAB strains was evaluated according to Özkan et al. [21] with minor modifications. The four selected *L. plantarum* isolates (M-1, SY-2, XJA2, XJ14) and the reference strain were subcultured twice and grown in MRS broth to the stationary phase. Cells were harvested by centrifugation, washed twice with sterile 0.85% (*w*/*v*) saline, and resuspended in 10 mL of sterile saline to achieve an initial optical density (OD_600_) of 1.0. After thorough vortexing, the suspensions were incubated statically at 37 °C. The absorbance of the upper suspension (*A*_t_) was measured at 600 nm using a UV-vis spectrophotometer (Cary 60, Agilent Technologies, Santa Clara, CA, USA) at 0, 4, 8, 12, and 24 h. The auto-aggregation percentage was calculated as follows:

$$Auto\text{-}aggregation\left(\%\right)=\left(1-\frac{A_{t}}{A_{0}}\right)\times 100\%$$

where *A*_0_ is the initial absorbance (0 h) and A_t_ is the absorbance at each sampling time.

#### 2.4.2. Prebiotic Utilization (GOS)

The ability of LAB strains to utilize prebiotics was determined following the method of Megur et al. [22] with slight modifications. To evaluate growth using galacto-oligosaccharides (GOS) as the sole carbon source, a modified MRS broth devoid of glucose (designated as MRS*) was used. Activated strains were washed twice with sterile 0.85% saline and resuspended in 1 mL of saline. The suspensions were inoculated into 9 mL of MRS broth (control) or MRS broth supplemented with 2% or 4% (*w*/*v*) GOS (Solarbio, Beijing, China). Cultures were incubated at 37 °C for 24 h, and optical density at 600 nm (OD_600_) was recorded every hour using an automated microbial growth analyzer (Bioscreen C, BioTek Instruments, Winooski, VT, USA). Growth was expressed as the net increase in OD_600_, calculated by subtracting the initial value from the final value.

#### 2.4.3. Hemolytic Activity Assay

Hemolytic activity was examined to assess the biosafety of LAB strains, using *Staphylococcus aureus* ATCC 25923 as a positive control for β-hemolysis. Each strain was activated twice and cultured to the stationary phase. A 100 μL aliquot of bacterial suspension was streaked on Columbia blood agar plates (Hopebio, Qingdao, China) supplemented with 5% (*v*/*v*) sterile defibrinated sheep blood. Plates were incubated at 37 °C for 48 h. Hemolysis was evaluated based on the zone surrounding colonies: β-hemolysis (clear halo), α-hemolysis (greenish halo), or γ-hemolysis (no visible change), according to the method described by Srinivas et al. [23].

#### 2.4.4. Biogenic Amine Production

Biogenic amine production was determined using the double-layer agar method, with *O. oeni* CEZ3 serving as the positive control. A 100 µL aliquot of stationary-phase culture was spread-plated onto the lower-layer agar containing 0.1% (*w*/*v*) amino acid precursors (tryptophan, arginine, lysine, tyrosine, phenylalanine, and histidine). After incubation at 37 °C for 48 h, a thin layer of indicator agar containing bromocresol purple (Solarbio, Beijing, China) was poured on top of the plate. The appearance of a purple color within 2 min indicated positive biogenic amine production, following the procedure outlined by Shiling et al. [24].

#### 2.4.5. Antibiotic Susceptibility Assay

Antibiotic susceptibility was determined using the E-test (MIC) method on MRS agar plates. The antibiotics tested included chloramphenicol, streptomycin, vancomycin, penicillin, and ampicillin (Kogene, Wenzhou, China). Activated bacterial cells were spread onto MRS agar plates and incubated at 37 °C for 18–24 h. Fresh colonies were then collected and suspended in sterile saline, and the turbidity of the suspension was adjusted to approximately 0.5 McFarland standard by visual comparison with a McFarland turbidity standard. The bacterial suspension was evenly spread over the entire surface of the agar plate. The E-test strips (bioMérieux, Marcy-l’Étoile, France) were placed vertically onto the agar surface. Plates were incubated statically at 37 °C for 16–20 h. The MIC value was read where the ellipse of inhibition intersected the strip. Results were interpreted according to the breakpoints established by the Clinical and Laboratory Standards Institute (CLSI).

### 2.5. Statistical Analysis

All experiments were performed in triplicate, and the results are expressed as mean ± standard deviation (SD). Prior to analysis, microbiological count data were log10-transformed. Statistical analyses were conducted using SPSS Statistics 26 (IBM, Armonk, NY, USA). Prior to analysis, the data were tested for normality using the Shapiro–Wilk test and homogeneity of variance using Levene’s test to ensure the assumptions of parametric testing were met. Subsequently, one-way analysis of variance (ANOVA) followed by Tukey’s multiple comparison test was employed to determine significant differences among strains. A *p*-value of less than 0.05 was considered statistically significant.

## 3. Results and Discussion

### 3.1. Primary Screening: Tolerance to Single-Factor Stresses

**Figure 1:**
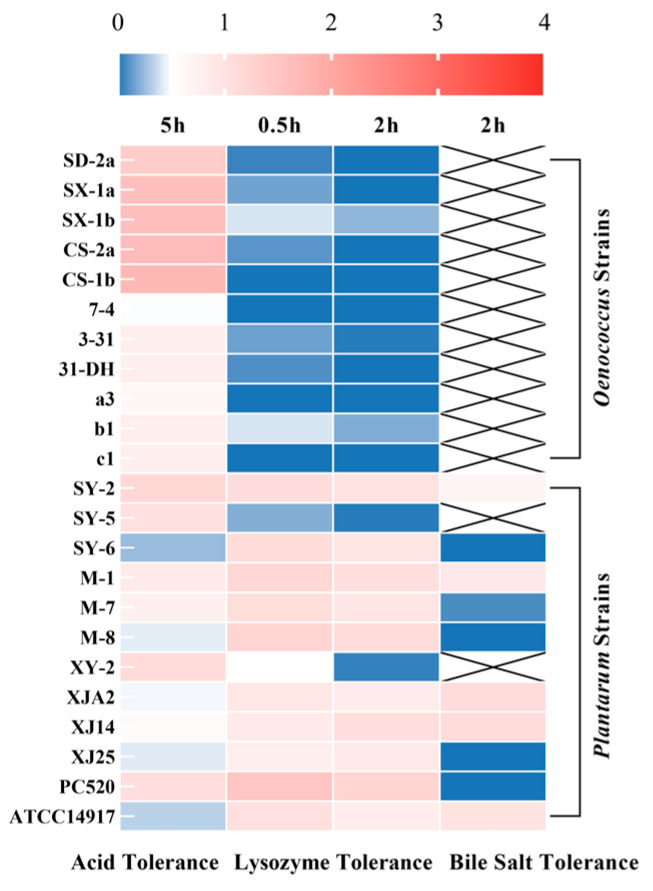
Screening of potential probiotic strains based on stress tolerance. Heatmap analysis representing the survival rates of *O. oeni* and *L. plantarum* strains under single-factor stress conditions. The heatmap color scale represents survival rates, with 50% defined as the critical threshold for selection (blue: low tolerance, survival < 50%; red: high tolerance, survival > 50%). Strains with crossed-out cells (×) indicate exclusion from that specific test due to failure in the preceding assay. Columns represent: Acid Tolerance: survival after 5 h at pH 3.2; Lysozyme Tolerance: survival after 0.5 h and 2 h in 100 mg/L lysozyme; Bile Salt Tolerance: survival after 2 h in 0.3% (*w*/*v*) bile salt.

Tolerance to acidity, lysozyme, and bile salts constitutes a fundamental prerequisite for microorganisms intended for probiotic applications. Accordingly, all 23 LAB isolates were first subjected to single-factor stress assays to establish baseline survival profiles prior to more integrative evaluations. The overall results, visualized as a heatmap (Figure 1), clearly reveal species-level trends and pronounced strain-level variability (*p* < 0.05). Numerical data are provided in Table S1.

#### 3.1.1. Acid Tolerance

Acid resistance represents a key, though not exclusive, criterion in probiotic screening. Gastric pH typically remains between 1.5 and 2.0 during fasting, but increases to approximately 3.2–3.5 following food intake [25]. Given that the reference strain *L. plantarum* ATCC 14917 retained only 33% viability at pH 3.2, the moderate, physiologically relevant postprandial pH was selected to enable meaningful comparison among isolates.

As illustrated in Figure 1, exposure to pH 3.2 for 5 h revealed substantial interspecific and intraspecific variation. All *O. oeni* isolates demonstrated exceptionally high acid tolerance (50–170%), markedly outperforming the reference strain (*p* < 0.05). This phenotype is consistent with the ecological adaptation of *O. oeni* to the acidic wine environment (pH 3.0–3.8) and aligns with previous studies highlighting its efficient proton-extrusion systems and membrane remodeling responses [15,26]. By contrast, *L. plantarum* isolates exhibited wide strain-dependent variation: while isolates such as SY-2, SY-5, XY-2, and PC520 maintained strong tolerance (>100%), others (SY-6, M-8, XJ25) showed pronounced sensitivity (<45%). These findings emphasize that acid tolerance alone is insufficient for preliminary selection, particularly given that downstream gastrointestinal stressors critically influence overall robustness.

#### 3.1.2. Lysozyme Tolerance

Lysozyme serves as one of the earliest host-defense barriers encountered by orally ingested microbes. Secreted primarily in saliva, this enzyme hydrolyzes β-(1,4)-glycosidic bonds in peptidoglycan, compromising bacterial cell wall integrity [27]. To simulate this oral-phase challenge, isolates were exposed to 100 mg/L lysozyme for 2 h, approximating physiologically relevant salivary concentrations [18].

As shown in Figure 1, the most striking observation was the extreme sensitivity of all *O. oeni* isolates to lysozyme. A pronounced decline in viability occurred within the first 0.5 h, and after 2 h of exposure, survival rates for all strains fell below 25% (*p* < 0.05). These results indicate that *O. oeni* is unable to withstand even moderate lysozyme pressure, despite its otherwise robust acid tolerance. This stark contrast highlights a fundamental limitation of this species as a probiotic candidate. The marked susceptibility of *O. oeni* likely reflects a relatively fragile cell wall architecture and limited defensive mechanisms against lysozyme; possible contributing factors include lower levels of peptidoglycan O-acetylation or altered autolysin regulation, although these mechanisms remain to be directly demonstrated [15,28]. Because *O. oeni* cannot overcome this initial oral enzymatic barrier, all 11 isolates were excluded from further characterization. In contrast, the *L. plantarum* isolates exhibited generally higher lysozyme tolerance, although the degree of resistance varied substantially among strains. Most isolates retained more than 80% viability after 2 h, reflecting the species’ inherently robust peptidoglycan layer and well-developed cell envelope stress responses. However, two strains, SY-5 and XY-2, showed extremely poor tolerance, with survival declining to 1.67% and 4.33% respectively—levels comparable to *O. oeni*. This pronounced intraspecies variation underscores the necessity for strain-specific evaluation rather than assumptions based solely on species-level traits. Similar heterogeneity in lysozyme resistance among *L. plantarum* strains has been reported previously [29]. Such variation is likely linked to differences in cell envelope composition and the efficiency of inducible stress response pathways.

Lysozyme tolerance therefore served as a decisive filtering step, eliminating all *O. oeni* isolates and identifying *L. plantarum* SY-5 and XY-2 as having critical vulnerabilities, which excluded them from the subsequent bile salt tolerance assay. These findings underscore the necessity of incorporating oral-phase stressors into early probiotic screening strategies.

#### 3.1.3. Bile Salt Tolerance

Bile salts represent one of the first major physiological stressors encountered by lactic acid bacteria upon entering the small intestine. Owing to their detergent-like properties, bile salts can disrupt cytoplasmic membrane integrity, alter protein conformation, and disturb intracellular homeostasis; thus, bile tolerance is widely regarded as a critical determinant in probiotic screening [30]. Considering that the bile salt concentration in the small intestine of healthy adults typically ranges between 0.1% and 0.3%, a physiologically relevant concentration of 0.3% (*w*/*v*) was employed in this study to evaluate the survival of the remaining ten *L. plantarum* isolates following 2 h of exposure [31].

As shown in Figure 1, the *L. plantarum* isolates displayed pronounced strain-dependent variability in their responses to bile stress (*p* < 0.05). A majority of strains, including M-1, SY-2, SY-5, XY-2, XJA2, and XJ14, maintained survival rates between 60% and 110% after 2 h of exposure, indicating robust tolerance. This observation is consistent with the versatile ecological nature of *L. plantarum* as a common intestinal commensal, which typically possesses well-developed bile resistance mechanisms, including bile salt efflux systems, bile salt hydrolase (BSH) activity, and membrane lipid remodeling [32,33]. In contrast, six isolates exhibited extremely poor or absent bile tolerance. Specifically, strains SY-6, M-8, XJ25, and PC520 showed complete loss of viability (0%), while M-7 (8%) and M-9 (23%) also displayed severely compromised survival—far below any physiologically meaningful threshold. These findings suggest that these strains lack essential bile resistance mechanisms. Previous studies have shown that, even within the same species, the presence, expression, or activity of BSH genes can vary markedly, contributing to substantial intraspecies heterogeneity in bile tolerance [34]. Importantly, bile tolerance serves as a more decisive exclusion criterion than acid tolerance. A strain that cannot survive bile exposure will be unable to remain viable upon reaching the small intestine, regardless of its favorable performance under other stressors such as acidity or lysozyme. Consequently, strains SY-6, M-7, M-8, M-9, XJ25, and PC520 should be excluded from all subsequent analyses. When considered together with lysozyme and acid tolerance results, the high degree of intraspecies variability in *L. plantarum* becomes even more apparent, with substantial inconsistencies across different stress conditions. For example, SY-6 tolerated lysozyme well but was completely inactivated by bile salts; similarly, PC520 exhibited excellent acid tolerance (>100%) but failed entirely under bile exposure. Such cross-stress inconsistency highlights the necessity of a multi-stage, multi-parameter screening funnel, as no single assay is sufficient to predict a strain’s overall robustness during gastrointestinal transit.

Overall, among the twelve *L. plantarum* isolates, only a subset consistently demonstrated adequate tolerance to bile salts. Strains exhibiting complete or near-complete sensitivity were identified as possessing critical deficiencies regarding probiotic application and therefore excluded from the subsequent simulated gastrointestinal assays. Taken together with lysozyme and acid tolerance data, these findings reinforce the importance of integrating multiple gastrointestinal stress parameters when identifying resilient probiotic candidates.

### 3.2. Secondary Screening: Simulated Gastrointestinal Tolerance

Following the single-factor assays, only four *L. plantarum* strains (M-1, SY-2, XJA2, and XJ14) demonstrated concurrent tolerance to acid, lysozyme, and bile salts. In contrast, all *O. oeni* isolates and two *L. plantarum* strains (SY-5 and XY-2) were removed due to failure in the lysozyme test, and six additional *L. plantarum* strains (SY-6, M-7, M-8, M-9, XJ25, and PC520) were eliminated due to failure in the subsequent bile salt test. These four strains were therefore advanced to secondary gastrointestinal simulations designed to mimic the integrated, multi-factor challenges encountered during in vivo digestion.

#### 3.2.1. Simulated Gastric Juice (SGJ)

**Figure 2:**
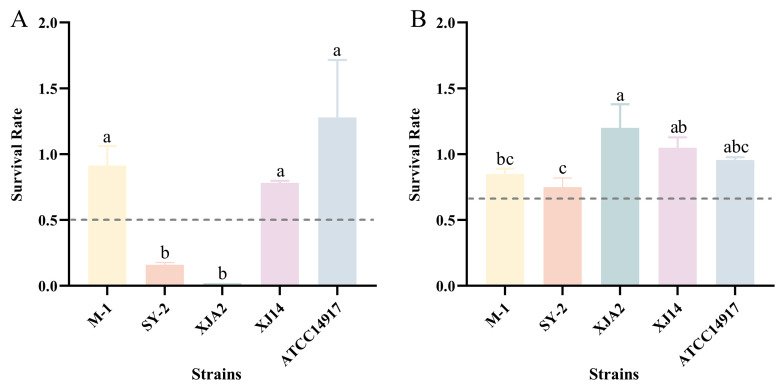
Gastrointestinal tolerance of superior *L. plantarum* strains. Strains were selected based on preliminary stress tolerance assays. (**A**) Survival rates in simulated gastric juice (SGJ, pH 3.0) after 3 h. (**B**) Survival rates in simulated intestinal juice (SIJ, pH 8.0) after 4 h. The dashed line indicates the 50% survival threshold. Data are expressed as mean ± SD (*n* = 3), with error bars representing the standard deviation. Different lowercase letters indicate significant differences (*p* < 0.05). ATCC 14917 was used as the control.

Exposure to SGJ (pH 3.0, containing pepsin) represents one of the most rigorous physiological barriers for oral probiotics, as the combination of acidity and proteolytic activity can rapidly compromise cell viability [35]. As shown in Figure 2A, significant strain-dependent differences were observed among the *L. plantarum* isolates. Strains M-1 and XJ14 demonstrated superior tolerance, with survival rates of 91% and 80%, respectively. These values were comparable to, though slightly lower than, the reference strain ATCC 14917 (128%). Their robust performance suggests the possession of effective acid-enzymatic tolerance mechanisms, consistent with the powerful stress response systems typically reported in robust *L. plantarum* genotypes [36]. In contrast, SY-2 (16%) and XJA2 (2%) displayed severe viability loss, despite having passed the single-factor assays in Section 3.1. This discrepancy underscores the limitations of isolated stress tests, as strain behavior under SGJ reflects the combined effects of acidity, ionic strength, and protease activity—conditions not captured by individual assays. Thus, SGJ exposure effectively differentiated strains with genuine gastric resilience from those with conditional or partial tolerance.

#### 3.2.2. Simulated Intestinal Juice (SIJ)

Tolerance to SIJ, characterized by alkaline pH and the presence of pancreatin and bile salts, constitutes the final major barrier prior to strain interaction with the host gut. As illustrated in Figure 2B, the overall performance of strains improved in SIJ, with some isolates achieving survival rates comparable to the reference strain. Strains XJA2, XJ14, and M-1 exhibited strong intestinal tolerance, with survival rates of 120%, 105%, and 85%, respectively, approaching that of the reference strain (96%). These results suggest that once the gastric bottleneck is bypassed, these isolates can effectively resist intestinal digestive enzymes and bile extracts. Similar observations have been reported in previous studies [37,38], which showed that certain *Lactiplantibacillus* strains, although highly sensitive to simulated gastric juice, exhibit strong survival under simulated intestinal conditions. This phenomenon indicates that tolerance to gastric acidity and resistance to intestinal stresses are not necessarily correlated and may depend on distinct strain-specific adaptive mechanisms. Strain SY-2 exhibited moderate tolerance (75%), which, although lower than the other three isolates, remains within a physiologically acceptable range. Despite the poor performance of SY-2 under SGJ conditions, its improved survival in SIJ indicates that its vulnerability is primarily associated with gastric stress rather than intestinal stress.

Overall, the simulated gastrointestinal assays identified *L. plantarum* M-1 and XJ14 as the only strains capable of maintaining high viability across both gastric and intestinal phases. Although the extremely low SGJ survival rates of XJA2 and SY-2 would typically warrant exclusion at this stage, it must be noted that the data in this study were obtained using unprotected cells directly exposed to an extremely acidic environment. In practical applications, probiotics are often consumed within food matrices such as dairy products or protected via modern delivery systems including microencapsulation [39,40]. These methods provide significant buffering capacity and mitigate direct gastric acid damage to the cells. Given the exceptional intestinal adaptation demonstrated by XJA2 and SY-2 after the gastric barrier is bypassed, particularly the strong performance of XJA2 with SIJ survival rates that exceeded those of the commercial control strain, removing these isolates at this stage could result in the loss of candidates with substantial intestinal colonization potential. Therefore, based on the premise that their gastric sensitivity can be overcome by formulation technologies, this study decided to retain these two strains were retained for subsequent functional evaluation. These findings underscore the importance of a multi-stage, multi-parameter screening workflow, indicating that resilience to individual stressors does not reliably predict the ability of LAB to survive physiologically relevant gastrointestinal transit.

### 3.3. Functional Characterization

Functional characterization was conducted on the four wine-derived *L. plantarum* strains (M-1, SY-2, XJA2, and XJ14) and compared against the reference strain ATCC 14917. Although SY-2 and XJA2 exhibited limited tolerance in the SGJ challenge, their strong performance in SIJ suggested that they may still retain traits relevant to intestinal persistence, particularly in realistic consumption scenarios involving food matrices or protective encapsulation. Therefore, the functional assessment focused on two key attributes associated with probiotic efficacy: auto-aggregation and utilization of galacto-oligosaccharides (GOS), which collectively contribute to competitive adhesion, ecological fitness, and host-microbe metabolic interactions [41,42].

#### 3.3.1. Auto-Aggregation Ability

**Figure 3:**
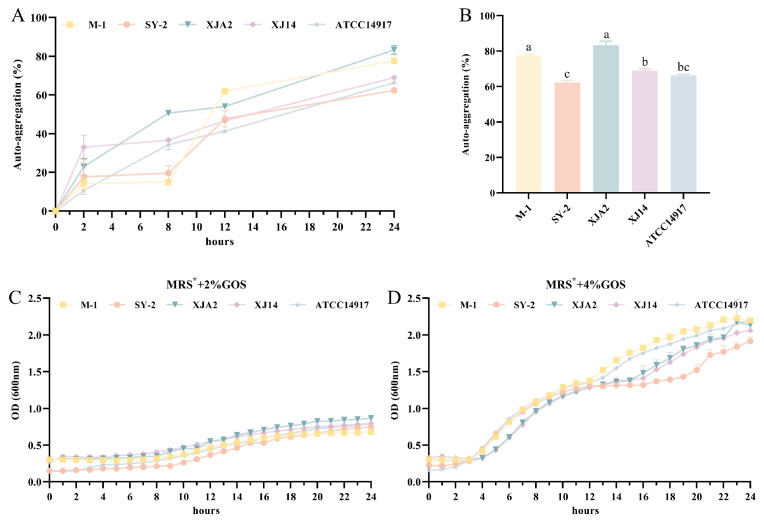
Functional probiotic properties: Auto-aggregation and prebiotic utilization. (**A**) Kinetic curves of auto-aggregation percentage over 24 h. (**B**) Final auto-aggregation rates at 24 h. (**C**,**D**) Growth curves (OD_600_) of strains cultured in MRS* broth (glucose-free) supplemented with different concentrations of GOS: (**C**) MRS* + 2% GOS, and (**D**) MRS* + 4% GOS. Error bars represent standard deviation (*n* = 3). Distinct letters in (**B**) indicate significant differences (*p* < 0.05).

Auto-aggregation reflects a strain’s capacity to self-associate into multicellular clusters, a phenomenon linked to mucosal adherence, competitive exclusion of pathogens, and the early steps of biofilm establishment in the gastrointestinal environment [43]. As illustrated in Figure 3A, all strains demonstrated a clear, time-dependent rise in auto-aggregation over the 24 h assay, a pattern consistent with the well-documented surface-associated traits of *L. plantarum*, which favor cell–cell adhesion and microcolony formation [44]. At 24 h (Figure 3B), XJA2 demonstrated the strongest auto-aggregation capacity (83.33%), significantly exceeding both the reference strain ATCC 14917 (66.33%) and SY-2 (62.33%) (*p* < 0.05). M-1 (77.76%) also exhibited robust aggregation, statistically comparable to XJA2 but significantly higher than the reference (*p* < 0.05). XJ14 showed an aggregation level of 69%, similar to the reference strain. Importantly, all strains surpassed the commonly accepted threshold of 40% auto-aggregation, a benchmark frequently used to indicate strong adhesion potential [45]. The consistently high values observed across these wine-derived isolates suggest the potential involvement of surface-associated factors often linked to aggregation, such as sortase-dependent proteins, exopolysaccharides, or cell surface hydrophobicity, which have been characterized in other adhesive *L. plantarum* genotypes [46,47,48]. These results further support the notion that wine-associated environments, characterized by nutrient limitation and intense microbial competition, may favor the enrichment of strains with strong aggregation and adhesion phenotypes.

#### 3.3.2. Utilization of Galacto-Oligosaccharides

Efficient utilization of prebiotic oligosaccharides is an important determinant of probiotic competitiveness and supports potential synbiotic interactions within the gut ecosystem [49]. To assess the ability of the selected isolates to metabolize GOS, growth was monitored in MRS* broth, which is a modified MRS formulation in which glucose was removed and replaced with GOS as the sole carbon source. As shown in Figure 3C, cultures grown in MRS* containing 2% (*w*/*v*) GOS exhibited only moderate growth, with final OD_600_ values remaining below 1.0. This indicates that GOS at this concentration cannot fully substitute for the 2% glucose present in standard MRS, possibly due to lower transport efficiency or the metabolic cost associated with inducing specific hydrolysis pathways compared to simple glucose metabolism [50]. When the GOS concentration was increased to 4% (Figure 3D), all isolates showed markedly improved growth and reached final OD_600_ values above 2.0. This clear dose-dependent response suggests that a higher substrate supply compensates for the metabolic costs of utilizing complex carbohydrates. Among the isolates, M-1, XJA2, and XJ14 demonstrated particularly rapid exponential-phase growth under the 4% GOS condition and achieved high cell densities. These findings indicate that the wine-derived *L. plantarum* isolates possess functional mechanisms involved in GOS metabolism, including oligosaccharide transport and β-galactosidase activity, which allow efficient utilization when sufficient substrate is provided [42]. From a probiotic perspective, the ability to ferment GOS is advantageous because it may enhance competitive persistence in the gut and contribute to the production of beneficial fermentation metabolites.

When evaluated together, the functional data indicate that M-1 and XJ14 exhibit the most balanced probiotic profiles, combining strong survival under gastrointestinal conditions with robust auto-aggregation and efficient GOS utilization. XJA2, although sensitive to simulated gastric juice, exhibited the highest auto-aggregation and excellent GOS metabolism, indicating strong functional potential that may be realized using protective delivery technologies such as encapsulation. SY-2 showed moderate aggregation and good GOS utilization, but its comparatively weak SGJ survival positions it below the other isolates in overall suitability. These findings highlight the multifaceted nature of probiotic screening: strains that excel functionally may not always excel physiologically, and vice versa. By integrating adhesion-related traits with metabolic adaptability, the functional characterization stage helps refine the selection of strains with the greatest potential for either direct or formulation-dependent applications.

### 3.4. Safety Assessment

Ensuring safety is an indispensable requirement for any microorganism intended for probiotic use in humans. Accordingly, the top-performing strains identified during functional screening (M-1 and XJ14), together with two representative isolates (SY-2 and XJA2), were subjected to a comprehensive safety assessment. The evaluation encompassed hemolytic activity, biogenic amine (BA) formation, and antibiotic susceptibility, parameters that collectively determine whether a strain poses potential risks to the host or carries traits that could disseminate undesirable characteristics within the gut microbiota.

#### 3.4.1. Hemolytic Activity

**Figure 4:**
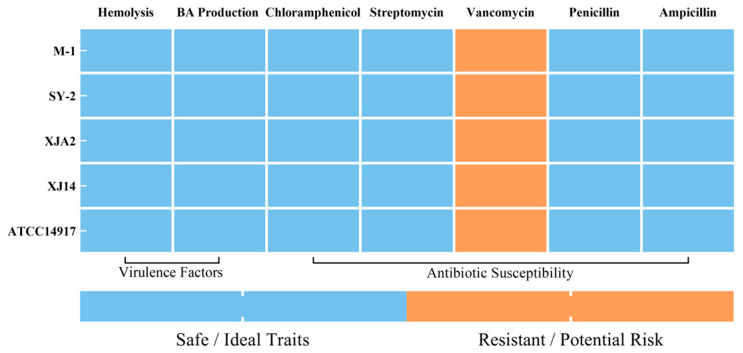
Comprehensive safety profile of the selected *L. plantarum* strains. The heatmap integrates the results of safety assessments in the following order: Hemolytic Activity, BA Production, and Antibiotic Susceptibility. In the color legend, Blue represents “Safe/Ideal Traits” (indicating γ-hemolysis, negative BA production, and antibiotic susceptibility), whereas Orange represents “Resistant/Potential Risk” (indicating antibiotic resistance). Note that the observed resistance to Vancomycin (orange columns) is intrinsic to the *Lactiplantibacillus* genus and chromosomally encoded, thus does not represent a transmissible safety hazard.

Hemolytic activity is a well-recognized virulence factor among pathogenic bacteria [51]. As summarized in the comprehensive safety heatmap (Figure 4), none of the tested *L. plantarum* isolates exhibited β-hemolysis (complete hemolysis) or α-hemolysis (partial hemolysis) when assessed on sheep blood agar. All strains displayed a γ-hemolytic phenotype, consistent with the behavior of the reference strain ATCC 14917. These findings indicate that the isolates lack hemolysin-encoding genetic determinants and meet the fundamental biosafety criterion expected for probiotic candidates. This finding is consistent with published safety assessments reporting non-hemolytic behavior for multiple *L. plantarum* strains [15,52].

#### 3.4.2. BA Production

The formation of biogenic amines, particularly histamine, tyramine, putrescine, and cadaverine, is a major safety concern, as excessive BA accumulation in fermented foods or the gastrointestinal tract may elicit adverse toxicological effects, including hypertension, palpitations, and headaches [53]. As illustrated in Figure 4, all tested *L. plantarum* isolates tested negative for BA production. This qualitative result indicates that the tested strains lack functional amino acid decarboxylase activity required for the biosynthesis of BAs. This observation aligns with established evidence that many food-associated *L. plantarum* strains either do not harbor key decarboxylase genes or display negligible decarboxylating activity [54,55].

#### 3.4.3. Antibiotic Susceptibility

Antibiotic susceptibility is critical not only for consumer safety but also for preventing the horizontal transfer of resistance genes within the gastrointestinal environment [56]. MICs for clinically relevant antibiotics, including chloramphenicol, streptomycin, vancomycin, penicillin, and ampicillin, were determined using E-tests and interpreted according to CLSI breakpoints.

As presented in Figure 4, all tested *L. plantarum* isolates were susceptible to chloramphenicol, streptomycin, penicillin, and ampicillin, with inhibition zones exceeding CLSI sensitivity thresholds. This suggests the absence of acquired resistance determinants and minimizes the risk of transferring undesirable traits to resident gut microbiota. All isolates exhibited resistance to vancomycin. Importantly, this phenotype is widely recognized as intrinsic to *L. plantarum*, rather than acquired within several *Lactobacillus* groups, particularly *L. plantarum*, *L. rhamnosus*, and *L. casei* [57]. Intrinsic vancomycin resistance in *Lactobacillus* is generally associated with chromosomally encoded features of the peptidoglycan synthesis pathway. In particular, the presence of a D-Ala-D-Lac terminus reduces the binding affinity of vancomycin. Although this mechanism is considered the predominant basis of intrinsic resistance, it is distinct from acquired resistance mediated by mobile genetic elements such as plasmids or transposons [58,59]. Therefore, the observed vancomycin resistance does not represent a biosafety hazard. Collectively, the absence of acquired antibiotic resistance, combined with the lack of hemolytic activity and biogenic amine production, confirms that isolates M-1, SY-2, XJA2, and XJ14 possess a favorable safety profile suitable for potential probiotic applications.

## 4. Conclusions

This study provided a systematic, multi-stage evaluation of lactic acid bacteria isolated from the spontaneous wine ecosystem and demonstrated that wine ecosystems constitute a valuable reservoir for probiotic candidates with distinctive stress-adaptation and functional advantages. Through a stepwise screening strategy, three *L. plantarum* isolates, namely M-1, XJ14, and XJA2, were identified as high-potential candidates. Among them, M-1 and XJ14 consistently exhibited strong survival across gastrointestinal simulations, robust auto-aggregation capacity, and efficient utilization of galacto-oligosaccharides, underscoring their suitability for direct incorporation into probiotic formulations. Although XJA2 showed limited tolerance to simulated gastric conditions, it displayed exceptional intestinal persistence and the strongest functional properties among the tested isolates, including superior auto-aggregation and high GOS-metabolizing capability. These traits suggest that XJA2 may serve as a functionally specialized strain whose full potential could be realized through protective delivery strategies such as microencapsulation or incorporation into food matrices that buffer gastric acidity. Comprehensive safety assessments confirmed that all evaluated isolates were non-hemolytic, produced no detectable biogenic amines, and exhibited antibiotic susceptibility profiles consistent with established safety criteria. Overall, this work highlights wine-derived *L. plantarum* strains as promising resources for developing next-generation probiotics. Future research should focus on in vivo validation to clarify their colonization behavior and health-promoting functions.

## Data Availability

The original contributions presented in this study are included in the article/Supplementary Materials. Further inquiries can be directed to the corresponding authors.

## Supplementary Materials

The following supporting information can be downloaded at: https://www.mdpi.com/article/10.3390/foods15061025/s1, Table S1: Survival Rates of LAB Strains under Single-Factor In Vitro Stress Conditions.
