# Supplementary material for: The Wine Ecosystem as a Reservoir for Potential Probiotics: A Comparative In Vitro Evaluation of Lactiplantibacillus plantarum and Oenococcus oeni Isolates
Source: Foods. 2026 Mar 15;15(6):1025. doi: 10.3390/foods15061025 (PMC13025300; doi:10.3390/foods15061025)
Supplement: Supplementary file 1 [file foods-15-01025-s001.zip › foods-4182290-supplementary.pdf]

Table S1. Survival Rates of LAB Strains under Single-Factor In Vitro Stress Conditions.

Strain	Species	Acid Tolerance (pH 3.2)	Lysozyme Tolerance (100 mg/L)		Bile Salt Tolerance (0.3% w/v)
		**5 h**	**0.5 h**	**2 h**	**2 h**
SD-2a	*Oenococcus oeni*	1.37±0.06^{b}	0.05±0.01^{ij}	0.00±0.00^{g}	N/A
SX-1a	*Oenococcus oeni*	1.57±0.06^{a}	0.16±0.03^{hi}	0.00±0.00^{g}	N/A
SX-1b	*Oenococcus oeni*	1.59±0.21^{a}	0.40±0.00^{g}	0.23±0.01^{f}	N/A
CS-2a	*Oenococcus oeni*	1.66±0.10^{a}	0.11±0.01^{hij}	0.00±0.00^{g}	N/A
CS-1b	*Oenococcus oeni*	1.70±0.17^{a}	0.00±0.00^{j}	0.00±0.00^{g}	N/A
7-4	*Oenococcus oeni*	0.49±0.09^{hi}	0.00±0.00^{j}	0.00±0.00^{g}	N/A
3-31	*Oenococcus oeni*	0.77±0.10^{efg}	0.15±0.05^{hij}	0.02±0.00^{g}	N/A
31-DH	*Oenococcus oeni*	0.79±0.04^{def}	0.09±0.01^{hij}	0.00±0.00^{g}	N/A
a_3	*Oenococcus oeni*	0.62±0.13^{fgh}	0.00±0.00^{j}	0.00±0.00^{g}	N/A
b_1	*Oenococcus oeni*	0.76±0.12^{efg}	0.40±0.04^{g}	0.19±0.03^{f}	N/A
c_1	*Oenococcus oeni*	0.76±0.03^{efg}	0.00±0.00^{j}	0.00±0.00^{g}	N/A
SY-2	*Lactiplantibacillus plantraum*	1.17±0.22^{c}	1.08±0.10^{bcd}	0.99±0.15^{bc}	0.68±0.04^{c}
SY-5	*Lactiplantibacillus plantraum*	1.01±0.05^{c}	0.20±0.02^{h}	0.02±0.00^{g}	N/A
SY-6	*Lactiplantibacillus plantraum*	0.25±0.02^{j}	1.09±0.09^{bcd}	0.94±0.03^{bcd}	0.00±0.00^{e}
M-1	*Lactiplantibacillus plantraum*	0.87±0.04^{de}	1.19±0.10^{bc}	1.07±0.04^{b}	0.90±0.02^{b}

M-7	*Lactiplantibacillus plantraum*	0.74±0.06^{efg}	1.07±0.15^{cd}	0.95±0.02^{bcd}	0.08±0.02^{e}
M-8	*Lactiplantibacillus plantraum*	0.43±0.05^{hij}	1.22±0.11^{b}	1.09±0.16^{b}	0.00±0.00^{e}
M-9	*Lactiplantibacillus plantraum*	0.71±0.02^{efg}	0.83±0.07^{f}	0.37±0.03^{e}	0.27±0.04^{d}
XY-2	*Lactiplantibacillus plantraum*	1.10±0.23^{c}	0.51±0.15^{g}	0.04±0.01^{g}	N/A
XJA2	*Lactiplantibacillus plantraum*	0.47±0.10^{hi}	0.91±0.09^{ef}	0.84±0.08^{cd}	1.10±0.19^{a}
XJ14	*Lactiplantibacillus plantraum*	0.58±0.07^{gh}	0.88±0.10^{f}	1.06±0.19^{b}	1.10±0.13^{a}
XJ25	*Lactiplantibacillus plantraum*	0.42±0.01^{hij}	0.80±0.09^{f}	0.86±0.01^{cd}	0.00±0.00^{e}
PC520	*Lactiplantibacillus plantraum*	1.06±0.02^{c}	1.48±0.07^{a}	1.24±0.25^{a}	0.00±0.00^{e}
ATCC14917	*Lactiplantibacillus plantraum*	0.33±0.04^{ij}	1.03±0.17^{de}	0.81±0.05^{d}	0.97±0.12^{ab}

Data are presented as mean ± standard deviation (n=3). Different lowercase superscripts (a, b, c...) in the same column indicate significant differences ($p < 0.05$). * N/A (not assessed): *Oenococcus oeni* strains were excluded from bile salt tolerance testing due to extremely low survival rates in the lysozyme tolerance assay.
